# Reproductive protein evolution in two cryptic species of marine chordate

**DOI:** 10.1186/1471-2148-11-18

**Published:** 2011-01-19

**Authors:** Marie L Nydam, Richard G Harrison

**Affiliations:** 1 Department of Ecology and Evolutionary Biology, Cornell University, Ithaca, New York 14853, USA

## Abstract

**Background:**

Reproductive character displacement (RCD) is a common and taxonomically widespread pattern. In marine broadcast spawning organisms, behavioral and mechanical isolation are absent and prezygotic barriers between species often operate only during the fertilization process. Such barriers are usually a consequence of differences in the way in which sperm and egg proteins interact, so RCD can be manifest as faster evolution of these proteins between species in sympatry than allopatry. Rapid evolution of these proteins often appears to be a consequence of positive (directional) selection. Here, we identify a set of candidate gamete recognition proteins (GRPs) in the ascidian *Ciona intestinalis *and showed that these GRPs evolve more rapidly than control proteins (those not involved in gamete recognition). Choosing a subset of these gamete recognition proteins that show evidence of positive selection (CIPRO37.40.1, CIPRO60.5.1, CIPRO100.7.1), we then directly test the RCD hypothesis by comparing divergence (omega) and polymorphism (McDonald-Kreitman, Tajima's D, Fu and Li's D and F, Fay and Wu's H) statistics in sympatric and allopatric populations of two distinct forms of *C. intestinalis *(Types A and B) between which there are strong post-zygotic barriers.

**Results:**

Candidate gamete recognition proteins from two lineages of *C. intestinalis *(Type A and B) are evolving more rapidly than control proteins, consistent with patterns seen in insects and mammals. However, ω (d_N_/d_S_) is not significantly different between the sympatric and allopatric populations, and none of the polymorphism statistics show significant differences between sympatric and allopatric populations.

**Conclusions:**

Enhanced prezygotic isolation in sympatry has become a well-known feature of gamete recognition proteins in marine broadcast spawners. But in most cases the evolutionary process or processes responsible for this pattern have not been identified. Although gamete recognition proteins in *C. intestinalis *do appear to evolve more rapidly, on average, than proteins with other functions, rates of evolution are not different in allopatric and sympatric populations of the two reproductively isolated forms. That sympatry is probably human-mediated, and therefore recent, may explain the absence of RCD.

## Background

Reproductive isolation between incipient species is of particular relevance to the process of speciation. Reproductive character displacement - 'the pattern of greater divergence of a (prezygotic) isolating trait in areas of sympatry between closely related taxa than in areas of allopatry' [1,2] is a common and taxonomically widespread pattern which is of great interest when studying reproductive isolation [3]. Evidence for RCD comes from groups as diverse as fungi [4,5], plants [6], insects [7,8] mollusks [9], fish [10,11] and amphibians [12]. However, the majority of RCD examples come from the *Drosophila *literature [13-17].

The study of RCD has historically been tied to the process of reinforcement, the evolution of prezygotic isolation resulting from selection against hybrid individuals [13-15]. More recently, however, workers have emphasized that RCD can be caused by other factors, including ecological variables [18,19]. But even where selection has been shown to play a role in RCD [20,21], the specific action of this selection remains unknown.

RCD has been documented in several taxa of marine broadcast spawning organisms [18]. The study of RCD is often more tractable in broadcast spawners than in internal fertilizers. Broadcast spawning individuals simultaneously release sperm and eggs into the water column, where fertilization occurs. Therefore, behavioral and mechanical isolation are absent in broadcast spawning organisms and if two sympatric broadcast spawning species are not temporally isolated [22], prezygotic barriers between these two species occur only during the fertilization process. Such barriers are usually a consequence of differences in the way in which sperm and egg proteins interact, so RCD is often manifest as faster evolution of these proteins between species in sympatry than allopatry [23,24]. Rapid evolution of these proteins appears to proceed through the action of positive (directional) selection, increasing the proportion of nonsynonymous substitutions (those that cause amino acid changes) relative to synonymous substitutions (the d_n_/d_s _ratio) [25].

New information has allowed us to use the broadcast spawning ascidian *Ciona intestinalis*, a longtime model in developmental biology, to study the early stages of RCD. *C. intestinalis *comprises two distinct and divergent lineages, now termed Type A and Type B [26-28], Type A is thought to be native to the Northwestern Pacific Ocean and to have invaded the Eastern Pacific Ocean, the Mediterranean Sea, the Atlantic coast of South Africa, and the Black Sea [29,30]. Type B, native to the Northern Atlantic Ocean [31,32], has invaded the Western Atlantic Ocean [27]. The ranges of Type A and B overlap in the English Channel and the Atlantic coast of France, where limited introgression occurs between Type A and B individuals from these sympatric populations [26]. Hybrids from crosses between allopatric individuals are sterile or inviable in the laboratory [28], but the evidence for introgression in nature shows that these two lineages are not completely reproductively isolated. Nothing is yet known about pre or postzygotic barriers in sympatry.

Here, we first identify a set of candidate gamete recognition proteins (GRPs) from *C. intestinalis *using proteomic and bioinformatic techniques and test whether these proteins evolve more rapidly than control proteins. Then, choosing a subset of these proteins that show evidence of positive selection, we directly test the RCD hypothesis by comparing divergence and polymorphism statistics in sympatric and allopatric populations of Type A and B *C. intestinalis*. We ask whether signatures of positive selection are stronger in sympatric than allopatric populations.

## Results

### Identification of candidate GRPs from sperm: proteomics

161 sperm proteins were identified by LC-MS/MS (Liquid Chromatography-Mass Spectrometry/Mass Spectrometry). Each of these proteins was subsequently identified using one or more of the following sections of the CIPRO database (*Ciona intestinalis *Protein): the descriptive summary available for many proteins, the Pfam Domain search, and the BlastP search, or the GO (Gene Ontology) program. 144 of the proteins are unlikely to be GRPs; they are likely involved in the movement or metabolism of the sperm (e.g. actin, dynein, myosin, tektin, α-tubulin, ATP-synthase, creatine kinase, enolase, malate dehydrogenase). The identities of these 144 proteins are available from the authors. Of the remaining 17 proteins, seven were likely GRPs, and 10 could not be identified as similar to any known proteins. Of these 17 proteins, four could not be analyzed because primers based on the Type A genome sequence would not amplify the homolog from Type B individuals. An additional gene could not be analyzed because the gene encoding the protein did not have significant tblastn hits to the Type A genome (and therefore no primers could be designed). We selected the remaining 12 proteins for further analysis, 3 of which were likely GRPs.

### Identification of candidate GRPs proteins from sperm: bioinformatics

We also used a bioinformatic approach to identify potential GRPs. First, we accessed the functional classifications of Type A testis ESTs sequenced by [33] and selected 25 ESTs that might code for GRPs. We then located the genes corresponding to these ESTs with the KOG (EuKaryotic Orthologous Groups) tool provided on JGI's (Joint Genome Institute) *C. intestinalis *genome browser http://genome.jgi-psf.org/Cioin2/Cioin2.home.html. These genes were then searched against the CIPRO database using blastx to identify resulting protein matches. 19 of these proteins were determined to be GRP candidates, but 10 failed to amplify and/or sequence in Type B individuals; the remaining nine were selected for further study.

Second, we located every protein in the CIPRO database that was identified as being expressed only in testes. We chose a subset of 10 of these proteins that were likely GRPs, of which nine could be amplified from Type B cDNA. In total, we selected 12 candidate GRPs identified proteomically and 18 identified bioinformatically. We also selected 9 control proteins (not involved in the fertilization process) from the proteomic analyses to compare with the putative GRPs. These control proteins, CIPRO129.24.1, CIPRO183.42.1, CIPRO2.134.1, CIPRO32.59.1, CIPRO53.35.1, CIPRO57.34.1, CIPRO68.37.1, CIPRO81.38.1 and CIPRO963.2.1, were selected because Pfam Domain and BlastP Searches in CIPRO identified them as proteins involved in basic cellular processes rather than fertilization (e.g. tubulin, NADH dehydrogenase, aconitate hydratase).

### Comparison of d_N_/d_S _values between candidate GRPs and control proteins

**Figure 1:**
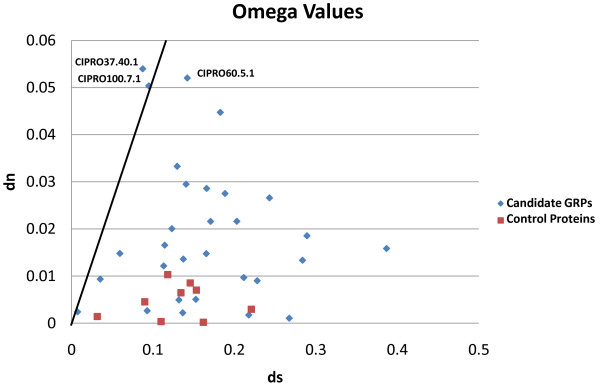
**Pairwise omega (d_N_/d_S_) values**. This graph shows pairwise d_N_/d_S _values (Type A vs. Type B *C. intestinalis*) for 30 candidate gamete recognition proteins (GRPs) and 9 control proteins. The line is d_N_/d_S _= 0.5.

The d_N_/d_S _values for candidate GRPs are significantly higher than the d_N_/d_S _values for control proteins (Figure 1, p = 0.004891 using a one-tailed Mann-Whitney U Test).

However, PAML analyses assume that d_S _values are constant across a sequence. If some sites across the sequence have unusually low d_S _values, a high d_N_/d_S _value could be inferred in the absence of positive selection [34]. Thus, significantly higher d_N_/d_S _values for candidate gamete recognition genes than for control genes could be the result of either higher d_N _or lower d_S _values in the candidate recognition genes, and only higher d_N _values provide evidence of positive selection.

To address this issue, we performed two-tailed Mann-Whitney U test in R (the Shapiro-Wilk test found evidence for non-normality), comparing d_N _values in candidate gamete recognition vs. control genes, and d_S _values in candidate gamete recognition vs. control genes. d_N _values were significantly different between candidate gamete recognition and control genes (p = 0.002), whereas d_S _values were not (p = 0.269). These tests are consistent with the assumption that d_N_, rather than d_S_, is driving the observed pattern.

Figure 1 also shows that two proteins have a d_N_/d_S _ratio greater than 0.5, which is the value above which we consider positive selection likely to be occurring when conservative pairwise d_N_/d_S _comparisons are used. A study by [35] across many different taxa showed that if a pairwise comparison yielded a d_N_/d_S _ratio of greater than 0.5, evidence for selection was subsequently observed when more sensitive site-specific tests were used. The rationale here is that d_N_/d_S _< 1 can still indicate positive selection at some subset of the amino acid residues within a protein, although much of the protein may be subject to constraint. Of the two proteins with d_N_/d_S _> 0.5, CIPRO37.40.1 was identified from the CIPRO database as having testis-only expression, and contains domains similar to ricin-type beta-trefoil lectin domains (d_N_/d_S _= 0.618, d_N _= 0.054, d_S _= 0.087).

CIPRO100.7.1 was identified proteomically, and its function is unknown. CIPRO100.7.1 is a large protein (1,225 amino acids), and was therefore divided into three sections for sequencing. Sections 2, 3, and all 3 sections analyzed together had d_N_/d_S _values less than 0.5, so only Section 1 was analyzed in subsequent sympatric vs. allopatric comparisons. Section 1 of CIPRO100.7.1 had a d_N_/d_S _equal to 0.531, a d_N _equal to 0.050, and a d_S _equal to 0.095. One protein, CIPRO60.5.1, has a d_N_/d_S _ratio lower than 0.5, but was identified in the CIPRO database as a metalloproteinase and has a GO biological function of "sperm binding to zona pellucida" (d_N_/d_S _= 0.366, d_N _= 0.052, d_S _= 0.142). We chose these three proteins for the sympatric vs. allopatric comparisons, either because they showed evidence of positive selection (in the case of CIPRO37.40.1 and CIPRO100.7.1), or because their putative function was so clearly related to gamete recognition (in the case of CIPRO60.5.1).

### Sympatric vs. allopatric divergence analyses

The results of the divergence analyses are shown in Table 1. For all of the candidate GRPs and control proteins, the 95% credible interval of the distribution of the differences between sympatric and allopatric ω (d_N_/d_S_) values included zero. This indicates that omega values were not significantly different between sympatry and allopatry for any of the proteins examined. Not enough variation was present in the sympatric Type A alleles for CIPRO60.5.1 or mtCOI to obtain reliable omega values, so the sympatric Type A vs. allopatric Type A comparison could not be performed for these proteins.

**Table 1:** Sympatric vs. allopatric comparisons of omega values

Candidate GRPs	Posterior mean of the difference between estimated omega values	95% credible interval of the difference between estimated omega values
CIPRO37.40.1 Sympatric A vs. Allopatric A	-0.52	-1.865 to 0.779
CIPRO37.40.1 Sympatric B vs. Allopatric B	0.01	-0.572 to 0.463
CIPRO60.5.1 Sympatric A vs. Allopatric A	NA (Not enough variation in sympatric Type A)	NA (Not enough variation in sympatric Type A)
CIPRO60.5.1 Sympatric B vs. Allopatric B	-0.09	-0.48 to 0.35
CIPRO100.7.1 Sympatric A vs. Allopatric A	0.29	-0.471 to 0.939
CIPRO100.7.1 Sympatric B vs. Allopatric B	0.01	-0.771 to 0.651
		
Control Proteins	Posterior mean of the difference between estimated omega values	95% credible interval of the difference between estimated omega values

CIPRO53.35.1 Sympatric A vs. Allopatric A	0	-0.015 to 0.0002
CIPRO53.35.1 Sympatric B vs. Allopatric B	0.19	-0.017 to 0.443
mtCOI Sympatric A vs. Allopatric A	NA (Not enough variation in sympatric Type A)	NA (Not enough variation in sympatric Type A)
mtCOI Sympatric B vs. Allopatric B	0.26	-0.064 to 0.791

### Sympatric vs. allopatric polymorphism analyses

The summary statistics are shown in Table 2. No consistent differences between sympatric and allopatric Type A or between sympatric and allopatric Type B can be discerned for any of these statistics for any of these candidate genes. Table 3 presents the results of the McDonald-Kreitman tests. Fixed nonsynonymous substitutions are not more common in sympatric than allopatric comparisons; these tests provide no evidence for positive selection on the genes encoding these three candidate GRPs in sympatry. None of the statistics for which significance was determined by coalescent simulation (D, D*, F*, H) showed significant differences between sympatry and allopatry for any of the genes examined, as the confidence intervals for sympatric populations always contained the mean values for allopatric populations, and vice versa (Table 4 for Tajima's D, Table 5 for D* and F*, Table 6 for H).

**Table 2:** Summary Statistics.

Candidate GRP genes	Population	n	θ	π (total)	π (synonymous sites)	π (nonsynonymous sites)
CIPRO37.40.1	Sympatric Type A	28	0.026	0.022	0.042	0.016
	Allopatric Type A	24	0.013	0.012	0.013	0.010
	Sympatric Type B	16	0.019	0.016	0.041	0.008
	Allopatric Type B	8	0.043	0.050	0.109	0.032
						
CIPRO60.5.1	Sympatric Type A	20	0.002	0.002	0.000	0.003
	Allopatric Type A	22	0.024	0.018	0.030	0.015
	Sympatric Type B	24	0.035	0.034	0.057	0.025
	Allopatric Type B	20	0.021	0.026	0.042	0.019
						
CIPRO100.7.1	Sympatric Type A	24	0.011	0.009	0.017	0.005
	Allopatric Type A	26	0.007	0.007	0.012	0.004
	Sympatric Type B	22	0.018	0.020	0.031	0.015
	Allopatric Type B	8	0.017	0.022	0.030	0.017
						
Control genes						

CIPRO53.35.1	Sympatric Type A	22	0.008	0.006	0.027	0.001
	Allopatric Type A	32	0.006	0.005	0.020	0.000
	Sympatric Type B	32	0.013	0.011	0.043	0.002
	Allopatric Type B	28	0.016	0.015	0.042	0.007
						
mtCOI	Sympatric Type A	10	0.000	0.000	0.000	0.000
	Allopatric Type A	12	0.006	0.004	0.021	0.000
	Sympatric Type B	9	0.005	0.005	0.023	0.000
	Allopatric Type B	11	0.005	0.005	0.021	0.001

n is the number of alleles sequenced from each population.

**Table 3:** Results of the McDonald Kreitman Tests for all genes.

Candidate GRP genes	Test	FS	PS	FN	PN	P value (Fisher's Exact Two Tailed Test)
CIPRO37.40.1	Sympatric Type A vs. Sympatric Type B	0	35	0	36	NA
	Allopatric Type A vs. Allopatric Type B	9	38	15	48	0.64
CIPRO60.5.1	Sympatric Type A vs. Sympatric Type B	6	27	7	22	0.76
	Allopatric Type A vs. Allopatric Type B	3	16	6	19	0.71
CIPRO100.7.1	Sympatric Type A vs. Sympatric Type B	9	25	12	30	1
	Allopatric Type A vs. Allopatric Type B	5	12	8	17	1
						
Control genes	Test	FS	PS	FN	PN	P value (Fisher's Exact Two Tailed Test)

CIPRO53.35.1	Sympatric Type A vs. Sympatric Type B	0	18	0	7	NA
	Allopatric Type A vs. Allopatric Type B	4	17	1	11	0.63
mtCOI	Sympatric Type A vs. Sympatric Type B	76	9	3	1	0.38
	Allopatric Type A vs. Allopatric Type B	70	21	3	2	0.59

FS = Fixed Synonymous. PS = Polymorphic Synonymous. FN = Fixed Nonsynonymous. PN = Polymorphic Nonsynonymous

**Table 4:** Tajima's D statistics for all genes

Candidate GRP genes	Population	D statistic	P-value	95% confidence interval
CIPRO37.40.1	Sympatric Type A	-0.078	0.281	"-1.68841 to 1.69923"
	Allopatric Type A	-0.004	0.157	"-1.20834 to 1.17"
	Sympatric Type B	-0.132	0.290	"-1.77 to 1.611"
	Allopatric Type B	0.006	0.092	"-0.99686 to 0.94395"
CIPRO60.5.1	Sympatric Type A	-0.018	0.541	"-1.51 to 1.66"
	Allopatric Type A	-0.087	0.561	"-1.69367 to 1.76737"
	Sympatric Type B	-0.058	0.528	"-1.45443 to 1.32636"
	Allopatric Type B	0.006	0.519	"-1.54796 to 1.72153"
CIPRO100.7.1	Sympatric Type A	-0.018	0.503	"-1.46162 to 1.35414"
	Allopatric Type A	0.009	0.510	"-1.3292 to 1.48969"
	Sympatric Type B	-0.014	0.515	"-0.92013 to 0.96708"
	Allopatric Type B	-0.042	0.526	"-1.35751 to 1.17643"
Control genes				

CIPRO53.35.1	Sympatric Type A	-0.057	0.544	"-1.68808 to 1.83296"
	Allopatric Type A	-0.002	0.511	"-1.37399 to 1.4227"
	Sympatric Type B	-0.027	0.524	"-1.42163 to 1.46487"
	Allopatric Type B	-0.061	0.545	"-1.31043 to 1.28035"
mtCOI	Sympatric Type A	-0.002	0.127	"-1.11173 to 1.43863"
	Allopatric Type A	-0.065	0.185	"-1.83094 to 1.77946"
	Sympatric Type B	-0.064	0.203	"-1.67754 to 1.75974"
	Allopatric Type B	-0.063	0.186	"-1.75914 to 1.8452"

**Table 5:** Fu and Li's D* and F* statistics for all genes

Candidate GRP genes	Population	D*	P-value	95% confidence interval	F*	P-value	95% confidence interval
CIPRO37.40.1	Sympatric Type A	-0.157	0.517	"-2.59 to 1.25985"	-0.111	0.496	"-2.498 to 1.52763"
	Allopatric Type A	-0.028	0.670	"-1.52677 to 1.239"	-0.063	0.589	"-1.58919 to 1.21221"
	Sympatric Type B	-0.034	0.578	"-2.24738 to 1.28647"	-0.090	0.540	"-2.66286 to 1.49659"
	Allopatric Type B	-0.024	0.734	"-1.00 to 0.87955"	0.007	0.612	"-1.03183 to 1.04566"
CIPRO60.5.1	Sympatric Type A	0.032	0.458	"-1.96617 to 1.25359"	-0.028	0.492	"-2.16055 to 1.477"
	Allopatric Type A	-0.040	0.432	"-2.464 to 1.25307"	-0.086	0.469	"-2.56615 to 1.49075"
	Sympatric Type B	-0.026	0.452	"-1.83617 to 1.17832"	-0.076	0.491	"-2.08 to 1.29"
	Allopatric Type B	-0.087	0.466	"-2.08275 to 1.17893"	-0.071	0.471	"-2.18321 to 1.38673"
CIPRO100.7.1	Sympatric Type A	-0.018	0.467	"-1.83829 to 1.22556"	-0.024	0.472	"-1.86692 to 1.35458"
	Allopatric Type A	-0.056	0.497	"-1.87585 to 1.23"	-0.070	0.506	"-2.09829 to 1.46478"
	Sympatric Type B	0.020	0.471	"-1.1613 to 0.99970"	-0.037	0.512	"-1.23262 to 1.02296"
	Allopatric Type B	-0.014	0.481	"-1.33924 to 1.16179"	-0.076	0.542	"-1.57039 to 1.29433"
Control genes							

CIPRO53.35.1	Sympatric Type A	-0.024	0.472	"-1.86692 to 1.35458"	0.013	0.456	"-2.20751 to 1.64394"
	Allopatric Type A	-0.070	0.506	"-2.09829 to 1.46478"	-0.012	0.501	"-1.68033 to 1.45582"
	Sympatric Type B	-0.037	0.512	"-1.23262 to 1.02296"	-0.057	0.504	"-1.91043 to 1.46351"
	Allopatric Type B	-0.076	0.542	"-1.57039 to 1.29433"	-0.004	0.474	"-1.88842 to 1.46422"
mtCOI	Sympatric Type A	0.008	0.762	"-1.24341 to 1.02623"	-0.039	0.786	"-1.34668 to 1.06879"
	Allopatric Type A	-0.006	0.440	"-2.229 to 1.40344"	-0.102	0.487	"-2.39875 to 1.54664"
	Sympatric Type B	-0.048	0.463	"-1.92508 to 1.43324"	-0.092	0.486	"-2.10684 to 1.58013"
	Allopatric Type B	-0.093	0.489	"-2.07471 to 1.42077"	-0.065	0.469	"-2.30147 to 1.50465"

**Table 6:** Fay and Wu's H test for all genes.

Candidate GRP genes	Population	H statistic	P-value	95% confidence interval
CIPRO37.40.1	Sympatric Type A	-0.166	0.348	"-21.476 to 7.34392"
	Allopatric Type A	-0.040	0.434	"-6.39 to 4.01449"
	Sympatric Type B	0.114	0.333	"-16.6833 to 6.3"
	Allopatric Type B	0.233	0.430	"-16.64 to 11.64"
CIPRO60.5.1	Sympatric Type A	0.006	0.351	"-2.37427 to 1.222"
	Allopatric Type A	-0.216	0.338	"-20.5 to 7.41126"
	Sympatric Type B	-0.144	0.394	"-18.956 to 9.159"
	Allopatric Type B	-0.161	0.392	"-13.53684 to 5.69474"
CIPRO100.7.1	Sympatric Type A	0.080	0.387	"-6.28261 to 3.78261"
	Allopatric Type A	0.014	0.401	"-4.61538 to 2.75692"
	Sympatric Type B	0.174	0.436	"-8.8658 to 6.02597"
	Allopatric Type B	-0.055	0.425	"-11.00 to 6.07143"
Control genes				

CIPRO53.35.1	Sympatric Type A	-0.061	0.347	"-5.36797 to 2.07792"
	Allopatric Type A	0.003	0.413	"-2.61694 to 1.54839"
	Sympatric Type B	0.017	0.420	"-4.03226 to 2.54839"
	Allopatric Type B	-0.027	0.406	"-6.68783 to 3.35979"
mtCOI	Sympatric Type A	-0.001	0.718	"-1.06667 to 0.4444"
	Allopatric Type A	0.024	0.312	"-6.72727 to 2.4242"
	Sympatric Type B	-0.044	0.344	"-6.47222 to 2.58333"
	Allopatric Type B	-0.119	0.315	"-8.69091 to 2.7272"

## Discussion

### Comparison of d_N_/d_S _values between candidate GRPs and control proteins

Candidate GRPs in *C. intestinalis *are evolving more rapidly than control proteins, and this pattern is likely driven by substitutions at nonsynonymous sites. Rapid evolution has been documented at specific GRPs (*i.e*. lysin, VERL and bindin) in marine broadcast spawners, and d_N_/d_S _values are lower for mtCOI than the GRPs lysin and VERL for green and pink abalone [36]. The pattern we see in *C. intestinalis *has also been documented in insects and mammals (e.g. butterflies: [37], field crickets: [38], mouse and human: [39], primates: [40]). This study suggests that a pattern of faster evolution in reproductive proteins may apply to external as well as internal fertilizers.

One explanation for faster evolution in candidate GRPs than control proteins is sperm competition, which occurs in *Ciona *as it does in internal fertilizers. Selection could be acting on any proteins that determine how quickly sperm fertilize eggs: proteins involved in metabolism, motility, binding, penetration, etc. However, as Figure 1 shows that candidate GRPs are evolving more rapidly than sperm proteins that are not candidate GRPs (control proteins), proteins directly involved in sperm-egg interactions are more likely to be experiencing directional selection than those involved in facilitating sperm access to the egg.

Rapid evolution of sperm GRPs might also result from sexual conflict, which occurs when the optimal outcomes of fertilization are different for sperm and eggs. For sperm, the optimal outcome is fertilization of an egg as quickly as possible. But in many taxa, fertilization of eggs by multiple sperm (polyspermy) results in developmental defects. Therefore, the optimal outcome of fertilization for an egg may be slower fertilization, to avoid polyspermy [25]. Ascidians like *C. intestinalis *often live in close proximity to many conspecific individuals [41], and an individual usually sends sperm into the water column before eggs (ascidians are hermaphrodites). So eggs are usually released into a vast amount of sperm spawned from many neighbors, making the risk of polyspermy very high. Perhaps in response to this risk, ascidians have evolved two separate blocks to polyspermy, whereas many other marine broadcast spawners have a single block [41]. Given these effective polyspermy blocks, sexual conflict resulting from polyspermy is not likely to be major driver of GRP evolution in ascidians.

Reinforcement could be driving the enhanced prezygotic isolation in sympatry. We know that hybrids between allopatric populations Type A and B *C. intestinalis *are sterile or inviable in the laboratory, so selection could favor GRPs that allow sperm to preferentially bind to conspecific eggs. However, reinforcing selection could be counteracted by gene flow from allopatric populations [42], especially when secondary contact is recent. Pairwise F_ST _calculations between sympatric and allopatric Type A, and sympatric and allopatric Type B populations show that these populations are not significantly differentiated at any of the three genes encoding the GRPs (data not shown); migration may therefore be occurring between sympatric and allopatric populations.

Lastly, egg surface proteins could be changing rapidly to prevent pathogens from entering the egg. If the same proteins involved in preventing microbial attack are involved in sperm/egg recognition, this could lead to the rapid evolution of sperm proteins to keep up with the ever-changing egg proteins.

### Evolution of candidate GRPs in *Ciona intestinalis *- no evidence for RCD

Our data provide no evidence that positive selection is enhanced in sympatry, and if these candidate GRPs are involved in prezygotic isolation, we have no evidence for enhanced prezygotic isolation. The polymorphism statistics likewise give no indication that RCD is occurring in these three proteins.

We cannot necessarily conclude from lack of evidence for RCD in CIPRO37.40.1, CIPRO60.5.1 and CIPRO100.7.1 that RCD is not occurring in this system. If enhanced prezygotic isolation between Type A and B does exist, there are several reasons why we might not have detected it in this study. First, primers for candidate GRPs were developed from the Type A genomic sequence and were used to amplify and sequence both Type A and B individuals (the Type B genome has not been sequenced). But Type A and B are substantially divergent (p-distances: 0.124 at mtCOI, 0.035 to 0.116 for six nuclear loci; [43]), which could explain why 15 genes encoding GRP candidates could not be successfully amplified and/or sequenced in Type B individuals. It is possible that the genes that could not be amplified and/or sequenced (and were therefore excluded from the analyses) encode proteins that are evolving more rapidly between Type A and B than those that were included in the analyses. If this is the case, we may have missed proteins for which d_N_/d_S _values are >1, proteins that would have been included in the sympatric vs. allopatric tests of RCD.

It is, of course, also possible that the three proteins that have high d_N_/d_S _values are themselves not involved in gamete recognition. While some aspects of the fertilization process in solitary ascidians such as *C. intestinalis *are well-characterized [44,45], the genes and corresponding proteins responsible for species-specificity have not been identified, as they have in other marine broadcast spawners [23,24].

Two sperm proteins that interact directly with the egg have been identified in *C. intestinalis*, but it is not known whether these proteins are involved in gamete recognition. The first protein is α L-fucosidase, which binds to the vitelline coat of the egg [46]. Five of our candidate GRPs (CIPRO187.4.1, CIPRO19.75.1, CIPRO33.15.1, CIPRO552.7.1, and CIPRO58.12.1) had domains also found in α L-fucosidases, but none of these proteins were expressed in testis tissue, based on expression data in CIPRO. CIPRO187.4.1 could not be amplified from Type B, and the other four genes did not have d_N_/d_S _ratios > 0.5. A second protein involved in sperm-egg interactions is a chymotrypsin-like enzyme that may dissolve the vitelline coat of the egg [47]. However, the amino acid sequence for this protein is not available and we identified dozens of chymotrypsin-like proteins in the genome.

Temporal isolation of Types A and B could also contribute to prezygotic barriers. We do not know whether Type A and B release gametes at the same time of day or in the same season in the English Channel. Since some gene flow has occurred [26], spawning must at least partially overlap.

Gene flow between sympatric and allopatric populations could be obscuring RCD if it is occurring in this system. Pairwise F_ST _calculations show that sympatric and allopatric populations of each type are not significantly differentiated, so gene flow between sympatric and allopatric populations is a possibility.

Finally, because secondary contact between Type A and B *C. intestinalis *in the English Channel may well be recent, RCD may not have yet taken place. Type B is native to Northern Europe and presumably a long-time resident of the English Channel. We do not know when Type A invaded the English Channel. The first published record of Type A in this area was in 2007 [26], but as Type A and B were only recognized in 2005 [48], Type A living in this area prior to 2005 would not have been distinguished from the native Type B. However, the introduction of Type A was likely human-mediated [26], and therefore a recent invasion on an evolutionary timescale. Evidence for RCD has been found in several systems where secondary contact is relatively recent. For example, RCD in mate choice is observed when limnetic and benthic sticklebacks co-occur in glacial lakes in British Columbia [20]. Similarly, RCD has been documented in the *Mus musculus *and *Mus domesticus *hybrid zone, which is thought to represent secondary contact during the Neolithic (since 9500 BCE) [49].

### Positive selection on GRPs in other marine broadcast spawners: RCD

Some of the most rapidly evolving proteins yet discovered are GRPs in marine broadcast spawners (*e.g*. bindin in sea urchins, lysin in abalone and mussels). In sea urchins, the bindin protein facilitates sperm attachment to the egg and fusion of sperm and egg [50]. In three genera of sea urchin that include sympatric species (*Echinometra, Heliocidaris*, and *Strongylocentrotus*), regions of bindin show evidence of positive selection ([51] and references therein). In *Arbacia, Lytechinus *and *Tripneustes*, genera that do not contain sympatric species, bindin shows no evidence of positive selection ([51] and references therein). This pattern is consistent with RCD on bindin in sea urchins.

Stronger evidence for RCD comes from a study of *Echinometra oblonga*, which has populations that are sympatric and allopatric with *Echinometra *species C [23]. Substantial divergence in bindin alleles between *E. oblonga *and *E*. sp. C. occurs where the two species are sympatric, but not where they are allopatric [23].

In abalone and mussels, sperm proteins known as lysins are involved in dissolution of the egg vitelline envelope, enabling the sperm to enter the egg. The best-characterized lysins are in the abalone genus *Haliotis*. An early study of 19 sympatric *Haliotis *species (19 sympatric and 1 allopatric species) found many pairwise comparisons with d_N_/d_S _values > 1 [52]. A later study of 25 species corroborated the pairwise results of [52] and also used maximum likelihood models of codon substitution to identify lineage and site-specific evidence of positive selection [53]. Lineages containing sympatric or closely related species usually had d_N_/d_S _values > 1, whereas lineages with distantly related allopatric species always had d_N_/d_S _values < 1, a pattern consistent with RCD [53]. But the authors also note a d_N_/d_S _value > 1 for the two branches separating a group of Japanese species from two groups of Californian species; this speciation event was likely allopatric.

In the mussel *Mytilus galloprovincialis*, two divergent clades of Lysin-M7 have been found: G and G_D _[24]. Evidence of positive selection is seen between G and G_D_, and within G_D _[24]. The divergence between the two clades is the result of rapid evolution in the G_D _clade, and G_D _alleles are found at higher frequencies in sympatric populations of *M. galloprovincialis *(where it hybridizes with *Mytilus edulis*) than in allopatric populations [24].

## Conclusions

Enhanced prezygotic isolation in sympatry has become a well-known feature of GRPs in marine broadcast spawners. But in most cases the evolutionary process or processes responsible for this pattern have not been identified. Differentiating between the processes that can lead to patterns of RCD will provide important insights into the process of speciation in marine broadcast spawners.

## Methods

### Identification of candidate GRPs from sperm: proteomics experiment

Sperm was collected from Type A individuals living in Santa Barbara, CA, filtered through a 70 μm nylon cell strainer (BD Biosciences) by centrifuging for 3 minutes at 3,000 rpm, and stored dry at -80°C. Sperm samples from several different individuals were later pooled and diluted 5-fold in phosphate buffer; the concentration of this dilution was determined to be 915 μg/ml. 500 μl of this diluted sperm was shipped to the University of Victoria Genome BC Proteomics Centre for the experiments described below.

9.5 M urea, 50 mM NH_4_HCO_3 _and 0.2% SDS were added to the sample, which was then sonicated. The proteins then underwent disulphide reduction and sulphydryl alkylation (200 mM DTT and 200 mM iodoacetamide) and were digested overnight at 37°C with 20 mg trypsin (Promega). Samples were subsequently cleaned with a cation exchange Cartridge Kit (Applied Biosystems).

#### Strong cation exchange chromatography

10 mM KH_2_PO_4 _(pH 2.7), 25% ACN buffer was added to the sample, which was then injected onto a Polysulphoethyl A strong cation exchange chromatography (SCX) column (PolyLC, Columbia, MD). The flow rate was set to 0.5 ml min^-1^. After equilibration, a 0-35% gradient of 10 mM KH_2_PO_4_, 25% ACN, 0.5 M KCl was added for 30 min. Each SCX fraction was then moved to autosampler vials (Dionex/LC Packings, Amsterdam).

#### One-dimensional reversed-phase chromatography with online mass spectrometry

A hybrid Quadruple-TOF LC-MS/MS mass spectrometer (QStar Pulsar I, MDS Sciex) with a nanoelectrospray ionization source (Proxeon, Odense, Denmark) was used to complete the liquid chromatography-mass spectrometry/mass spectrometry (LC-MS/MS) analyses. A C18AQ Nano LC and a Zorbax C18 guard column (Agilent Technologies) performed the chromatographic separation. The ANALYST QS v. 1.1 software service pack (ABI MDS SCIEX, Concord, Canada) gathered the data.

#### Mass spectrometry data analyses

the information dependent acquisition file was viewed using ANALYST v. 1.1 software, and the peak lists were assembled with a built-in MASCOT script (1.6b16 ABI--Matrix Science Limited). Spectra with less than 10 peaks were discarded. MASCOT v. 2.0 (Matrix Science Limited) was used to analyze the data. Spectrometry data were searched against a database of amino acid sequences from the CIPRO (*Ciona intestinalis *Protein) database http://cipro.ibio.jp/2.5/.

### Comparison of d_N_/d_S _values between candidate GRPs and control proteins

Of proteins identified by proteomic analysis, we selected 39 proteins (30 candidate GRPs and 9 control proteins) for further analysis using proteomic and bioinformatic approaches (see Results section). Genomic sequences for the genes encoding the 39 proteins were located by performing tblastn searches to the *Ciona intestinalis *Ensembl genome server http://uswest.ensembl.org/Ciona_intestinalis/Info/Index. Primers were developed in coding regions, and the partial or entire coding regions of all 39 proteins were sequenced from cDNA of two Type A and two Type B individuals, all from allopatric populations. Testis tissues from these four individuals were collected in 2008 and immediately placed in RNAlater (Ambion). The tissue/RNAlater was frozen at -80°C within seven days of collection.

Total RNA was extracted from testis tissue with the RNAdvance Kit (Agencourt) and was used to synthesize single-stranded cDNA using SuperScript III reverse transcriptase (Invitrogen) and an oligo (dT) primer. A 5-fold dilution of the single-stranded cDNA was then PCR-amplified with TRsa and TS-PCR primers. The resulting PCR product was diluted 50-fold and used as the template for amplification of the coding regions for the 30 candidate GRPs proteins and 9 control proteins. The amplified coding regions were incubated with 0.25 μl each of Exonuclease I and Shrimp Antarctic Phosphatase at 37°C for 30 min, followed by 90°C for 10 min. The products were purified using CleanSeq beads (Agencourt), and the purified products were sequenced with a Big Dye Terminator Cycle sequencing kit and an Automated 3730 DNA Analyzer (Applied Biosystems). Sequences were edited, trimmed and aligned with Aligner (CodonCode Corporation, Dedham, MA). Primers and cycling conditions are available from the authors.

Once the sequences were obtained, the codeml program in PAML 4.4 [54] was used to obtain pairwise d_N_/d_S _values for each Type A vs. Type B combination (Type A Individual #1 vs. Type B Individual #1, Type A Individual #1 vs. Type B Individual #2, Type A Individual #2 vs. Type B Individual #1, Type A Individual #2 vs. Type B Individual #2). The average d_N_/d_S _value for all four combinations was calculated for each gene, and the d_N_/d_S _values of the putative GRPs and control proteins were found to be distributed non-normally using the Shapiro-Wilk test in R (version 2.10.1). The Shapiro-Wilk test is the most robust test of non-normality for small to medium sample sizes [55,56]. The d_N_/d_S _values of the candidate GRPs and control proteins were therefore compared using a one-tailed Mann-Whitney U test in R (version 2.10.1).

### Sympatric vs. allopatric divergence analyses

Samples were collected in 2005-2009 from allopatric and sympatric populations of Type A and Type B. The allopatric population of Type A was from Half Moon Bay, CA while sympatric populations of Type A were from Perros-Guirec, France and Concarneau, France. Allopatric populations of Type B were collected from Woods Hole, MA and Gosport, England.

To obtain genomic DNA, ovaries were dissected from freshly-collected individuals, cut into several pieces, immediately preserved in DMSO (dimethyl sulfoxide), and ultimately (within 10 d) stored at -80°C until needed. Total DNA was extracted from the ovaries using the Qiagen DNeasy^® ^Tissue Kit (Qiagen Corporation, Santa Clarita, CA).

At least 10 individuals from each of 4 populations (allopatric Type A and B, sympatric Type A and B) were sequenced for the genes encoding three candidate GRPs: CIPRO37.40.1, CIPRO60.5.1, CIPRO100.7.1. The criteria for selecting these three candidate GRPs for the sympatric/allopatric comparison, from the 30 candidate GRPs used in the comparison of d_N_/d_S _values between GRPs and control proteins, are discussed in the Results section.

The same individuals were also sequenced for two control proteins: CIPRO53.35.1 and mitochondrial cytochrome oxidase I (mtCOI). Sequences from all five loci were deposited in GenBank (Accession Numbers HQ872081-HQ872454). A signature of enhanced selection in sympatry vs. allopatry could be due to selective processes or demographic processes (e.g. recent population growth). But demographic processes would affect all genes, not just candidate GRPs. So proteins not involved in the fertilization process (control proteins) were also subjected to divergence analyses. CIPRO53.35.1 was selected as a control protein because it contains a domain similar to a ribosomal L32 protein domain and is expressed in many different tissues; whereas mtCOI is an enzyme in the electron transport chain of the mitochondria. For all nuclear genes the PCR product for each locus was cloned using the pGEM^®^-T kit and up to eight clones were PCR-amplified and sequenced as described above to obtain both alleles. When only one allele was found in eight clones, the individual was assumed to be homozygous for that allele.

We used omegaMap version 0.5 [57] to determine whether omega values were significantly different for sympatric Type A vs. allopatric Type A populations and for allopatric Type A vs. allopatric Type B populations. We chose omegaMap over PAML for three reasons. First, omegaMap is designed to calculate omega values in the presence of recombination, and every one of the five genes (those encoding CIPRO37.40.1, CIPRO60.5.1, CIPRO100.7.1, CIPRO53.35.1, and mtCOI) has at least one population where intragenic recombination is present. Second, unlike PAML, omegaMap takes a population genetics approach, so we can use all the alleles we sequenced from each population in our calculation of omegaMap. Lastly, the Bayesian inference implemented in omegaMap provides a perfect framework for testing whether omega values are significantly different in sympatry vs. allopatry.

Using omegaMap, we calculated omega (d_N_/d_S_) values for sympatric Type A, sympatric Type B, allopatric Type A and allopatric Type B populations separately for each gene. We chose 250,000 iterations for each run, with thinning set to 1,000. We used an improper inverse distribution to specify priors for all parameters. Initial parameter values were μ = 0.1, κ = 3.0, ω = 0.5, ρ = 0.1. A constant model was used, so that all sites are assumed to have the same omega value. The number of iterations discarded as burnin varied across runs, but was determined by plotting the traces of μ and κ; iterations affected by the starting value of the parameter were discarded. Two independent runs were conducted for each population/gene (e.g. CIPRO37.40.1_Sympatric A Run 1 and 2). These two runs were combined in all cases, after it was determined that the mean and 95% credible interval for each parameter in the two runs matched closely.

After the omega values for each population/gene were estimated, we permuted the posterior distribution of CIPRO37.40.1 sympatric Type A omega values and the posterior distribution of CIPRO37.40.1 allopatric Type A omega values (for example), obtained a distribution of the differences between these values, and calculated the mean and 95% credible interval for this distribution. If the 95% credible interval contained zero, the estimated omega values were not significantly different. All calculations were done in R (version 2.10.1).

### Sympatric vs. allopatric polymorphism analyses

Both alleles from at least 10 individuals from each of four populations (allopatric Type A and B, sympatric Type A and B) were sequenced for three candidate gamete recognition genes and two control genes as described in the "Sympatric vs. allopatric divergence analyses" section.

For each population and each gene, the summary statistics θ and π were calculated in DnaSP 5.10.1 [58]. We also employed the following tests: McDonald-Kreitman [59], Tajima's D [60], Fu and Li's D* and F* [61], and Fay and Wu's H [62] in DnaSP. Statistical significance of D, D*, F* and H were determined using 1,000 coalescent simulations in DnaSP. Estimates of per gene recombination for each population were made in DnaSP and were then imported into the simulations. 95% confidence intervals for D, D*, F* and H statistics were also recorded; sympatric and allopatric populations were determined to be significantly different for each statistic if the confidence intervals of the sympatric population did not contain the mean of the allopatric population.

## Authors' contributions

MLN collected the samples, generated the data, performed the analyses and wrote the manuscript. RGH provided funding, and assisted with the design of the study and the editing of the manuscript. Both authors have read and approved the final manuscript.
